# Genome sequence of a Tetraparvovirus ungulate 1 strain from a cow in Tennessee, USA

**DOI:** 10.1128/mra.01291-24

**Published:** 2025-03-25

**Authors:** Ola K. Elsakhawy, Ashkan Roozitalab, Mohamed A. Abouelkhair

**Affiliations:** 1 Diagnostic Medicine and Pathobiology, Shreiber School of Veterinary Medicine, Rowan University 3536, Glassboro, New Jersey, USA; 2 Department of Biomedical and Diagnostic Sciences, University of Tennessee 204922, Knoxville, Tennessee, USA; Katholieke Universiteit Leuven, Leuven, Belgium

**Keywords:** respiratory, parvoviruses, bovine, metagenomics, Tetraparvovirus ungulate 1

## Abstract

We report the genome sequence of a strain belonging to the species Tetraparvovirus ungulate 1 (hereafter referred to as UTPV1-1882), identified in a 4-month-old cow with respiratory disease in Tennessee, USA. The sequence data will improve the existing genomic database, which currently lacks information on these viruses in the United States.

## ANNOUNCEMENT

Tetraparvoviruses, members of the *Parvoviridae* family, are small, non-enveloped viruses with a single-stranded DNA genome between 4,000 and 6,000 nucleotides in length (1). Their genetic material is organized into two open reading frames (1, 2). Tetraparvovirus ungulate 1, also known as bovine hokovirus and Ungulate tetraparvovirus 1 (UTPV1), was initially identified in domestic cattle from local food markets in Hong Kong (3, 4). Subsequently, it was discovered in apparently healthy yaks in the Guansu and Qinghai regions of China (5). Reports also indicate that UTPV1 was detected in 2016 in asymptomatic and sick steers from Mexico and the United States, as well as in cattle in South Brazil (6, 7). The genome of UTPV1/AB has been sequenced from a beef-suckler calf suffering from bovine respiratory disease in the East of Ireland (8). In October 2023, another UTPV1 strain was identified in diseased cattle from Hunan province, China (2). Metagenomic sequencing improves the diagnostic performance for respiratory infections by allowing unbiased detection of a wide range of pathogens (9, 10).

Lung tissue was collected from a 4-month-old euthanized cow with respiratory disease in 2023 and tested using an in-house respiratory diagnostic qPCR panel at the University of Tennessee College of Veterinary Medicine (UTCVM) diagnostic laboratory. The panel, which tests for bovine respiratory syncytial virus, bovine viral diarrhea virus, and infectious bovine rhinotracheitis, was negative. For sequencing, the sample was thoroughly homogenized by a FastPrep-24 homogenizer (MP Biomedicals, USA). DNA was extracted and purified using the MagMAX Viral/Pathogen Nucleic Acid Isolation Kit (Thermo Fisher Scientific, USA). The DNA quantity and quality were evaluated using a NanoDrop 2000 (Thermo Fisher Scientific, USA) and Qubit fluorometer (Fisher, Waltham, MA, USA). DNA was removed by dsDNase treatment using heat-labile Double-Strand Specific DNase (Thermo Fisher Scientific, USA) followed by inactivation at 58°C for 5 min in the presence of 10 mM DTT. For the second strand cDNA synthesis, the NEBNext Ultra II Directional RNA Second Strand Synthesis Module (New England Biolabs, MA, USA) was used following the manufacturer’s instructions. Sequencing libraries were prepared using the Nextera DNA Flex kit (Illumina, Inc., USA) according to the manufacturer’s instructions. Paired-end 2 × 150 bp sequencing was performed on an Illumina Miniseq. Metagenomic data analysis was performed using the Sunbeam pipeline (version 4.7.0) (11). Taxonomic classification was performed using Kraken2 on Illumina reads (12, 13). Then, viral reads were assembled into contigs using MEGAHIT (version 1.2.9) (14, 15). Out of the total 5,620 viral reads analyzed, a substantial proportion of 5,523 reads (98.27%) were classified as parvoviruses. The obtained genome of UTPV1-1882 was 5,226 nucleotides in size (GC content: 48.95%) and was annotated using Viral Annotation Pipeline and iDentification (version 1.2) (16). UTPV1-1882 showed identities of 88.6%–99.8% to the known Tetraparvovirus ungulate 1 strains from GenBank (Table 1). All the sequence analysis described in this study was conducted utilizing the Jetstream2 cloud computing resource (17). Default parameters were used except where otherwise noted.

**TABLE 1:** Publicly available genome sequences of Tetraparvovirus ungulate 1 strains

Accession	Strain/isolate	Organization	Length (bp)	Completeness	% identity of UTPV1-1882 to each strain	Country
NC_038898.1	HK1	National Center for Biotechnology Information, NIH	5,105	Complete	90.5	China
NC_028136.1	Yak hokovirus GS1	National Center for Biotechnology Information, NIH	5,111	Complete	90.3	China
PP541714.1	HNU-CBY-2023	Hunan University, College of Biology	5,346	Complete	97.2	China
OP113956.1	ABGEBB-83	Teagasc, Animal Bioscience Building	5,227	Partial	98.1	Ireland
OP020140.1	BoHoko2_NSW_L14	University of Technology Sydney, Australian Institute for Microbiology & Infection	3,969	Partial	99.5	Australia
MZ502236.1	BCS1/USA	Oklahoma State University, Veterinary Pathobiology	5,097	Partial	99.8	USA
MH814981.1	YN-BPV	Wuhan Institute of Virology, Chinese Academy of Sciences, State Key Laboratory of Virology	5,240	Complete	98.0	China
MG745679.1	Guarapuava	University of Sao Paulo, Virology Research Center	5,368	Partial	97.2	Brazil
KU172423.1	BS-S13	Kansas State University, Veterinary Diagnostic Laboratory and Department of Diagnostic Medicine/Pathobiology	5,254	Partial	98.8	USA
KT225725.1	Yak hokovirus GS1	Gansu Agricultural University, College of Veterinary Medicine	5,111	Complete	90.3	China
KT225726.1	Yak hokovirus GS2	Gansu Agricultural University, College of Veterinary Medicine	5,111	Complete	88.6	China
KT225727.1	Yak hokovirus QH1	Gansu Agricultural University, College of Veterinary Medicine	5,111	Complete	88.6	China
KT225728.1	Yak hokovirus QH2	Gansu Agricultural University, College of Veterinary Medicine	5,111	Complete	90.1	China
KT225729.1	Yak hokovirus QH3	Gansu Agricultural University, College of Veterinary Medicine	5,111	Complete	89.9	China
KT225730.1	Yak hokovirus QH4	Gansu Agricultural University, College of Veterinary Medicine	5,111	Complete	89.8	China
JF504697.1	HK-B15	The University of Hong Kong, Microbiology	5,096	Partial	90.4	China
JF504698.1	HK-B38	The University of Hong Kong, Microbiology	5,240	Partial	98.0	China
EU200668.1	HK3	The University of Hong Kong, Department of Microbiology	5,088	Partial	90.4	China
EU200669.1	HK1(2008)	The University of Hong Kong, Department of Microbiology	5,105	Partial	90.5	China
EU200670.1	HK2	The University of Hong Kong, Department of Microbiology	5,104	Partial	90.5	China

## Data Availability

This Whole Genome Shotgun project has been deposited in GenBank under accession no. PQ561209. The version described in this paper is the first version, PQ561209.1, and in the Sequence Read Archive under accession numbers SRR31204723.
